# *Indigofera suffruticosa* Mill. (Anil): Plant Profile, Phytochemistry, and Pharmacology Review

**DOI:** 10.1155/2018/8168526

**Published:** 2018-12-02

**Authors:** Janaina K. L. Campos, Tiago F. da S. Araújo, Thaíse G. da S. Brito, Ana P. S. da Silva, Rebeca X. da Cunha, Mônica B. Martins, Nicácio H. da Silva, Bianka S. dos Santos, César A. da Silva, Vera L. de M. Lima

**Affiliations:** ^1^Universidade Federal de Pernambuco (UFPE), Núcleo de Ciências da Vida, Centro Acadêmico do Agreste, Laboratório Morfofuncional, Rodovia BR 104, Km 62, S/N-Nova Caruaru, Caruaru, PE 55014-908, Brazil; ^2^Universidade Federal de Pernambuco (UFPE), Departamento de Bioquímica, Av. Prof. Moraes Rego, 1235-Cidade Universitária, Recife, PE 50670-901, Brazil; ^3^Universidade Federal do Vale do São Francisco, Colegiado de Farmácia, Av. José de Sá Maniçoba, S/N-Centro, Petrolina, PE 56304-917, Brazil; ^4^Universidade Federal do Vale do São Francisco, Colegiado de Medicina, Av. José de Sá Maniçoba, S/N-Centro, Petrolina, PE 56304-917, Brazil

## Abstract

*Indigoferasuffruticosa* Mill. (Fabaceae) is known as anil or anileira and also with other names, due to the production of a blue pigment, which is commonly used for yarn dyeing. It is distributed in some states of Brazil (Pernambuco, Paraíba, Mato Grosso, São Paulo, Bahia, Pará, and others) and is used in the popular medicine as a febrifuge, antispasmodic, diuretic, abortive, analgesic, purgative, or soothing agent against stomach and urinary problems, jaundice, and ulcers and also as an insecticide. In addition, *I. suffruticosa* can be used as animal feed. This review aimed at providing important data on the botanical, distribution, ethnopharmacology, phytochemical, pharmacological, and toxicity of *I. suffruticosa* based on the scientific literature. Information on *I. suffruticosa* was gathered via the Internet (from Elsevier, NCBI, and Sci-Hub) and libraries in the period from February to March 2016. More than 40 chemical compounds have been identified and a few compounds isolated, and the main origins are the essential oils, organic extracts, and aqueous extracts of different parts of the plant. *I. suffruticosa* and its active compounds possess wide pharmacological actions in the literature, such as anti-inflammatory, antibacterial, antifungal, antioxidative, antitumor, antimutagenic, anticonvulsant, gastroprotective, and hepatoprotective activities. Therefore, as an important traditional popular medicine, further studies on *I. suffruticosa* are required for the development of new drugs and therapeutics for various diseases.

## 1. Introduction

Fabaceae, present in the Brazilian biodiversity, is considered the third largest family of plants, which has about 19,500 species [1], and it is divided into three subfamilies: Mimosoideae, Caesalpinioideae, and Papilionoideae, and it shows a common feature in almost all fruits and vegetables, known as pods [2]. Papilionoideae is a subfamily with greater wealth in the Caatinga. Among diverse species, the *Indigofera* species is taxonomically present [3].

This family is considered of great importance because among the several varieties, many species are used for food purposes and is used as animal feed, latex, resins, raw materials in the manufacture of paints, pesticides, and medicinal drugs, before undergoing processing and purifications (*Dioclea megacarpa*, *Vatairea paraensis*, and *Dipteryx punctata*), and ornamental trees. Examples of species used as food sources are chickpea (*Cicer arietinum*), peas (*Pisum sativum*), beans (*Phaseolus vulgaris*), lentil (*Lens cultivaris*), and soybean (*Glycine max*) [4].

The genus *Indigofera* belonging to the Fabaceae family stands out for being used as fodder [5], green manure, and ground cover [6]. This genus has about 700 species distributed in Asia, tropical Africa, Australia, and North and South America; in Brazil, it is possible to find three species with same popular name “anileira”: *Indigofera truxillensis*, *I. hirsuta*, and *I. suffruticosa* [7].

A few decades ago, the investigations of *I. suffruticosa* have focused on their biological activities, including their antitumor [8], anti-inflammatory [9], antimicrobial [10, 11], and antiepileptic [12] properties, but now scientific studies have diversified and deepened their knowledge about this species. These studies evaluated the biological potential of different parts of the plant, with chemical compounds from extractions isolated by using various solvents. This review aimed at providing important data on the botanical, distribution, ethnopharmacology, phytochemical, pharmacological, and toxicity of *I. suffruticosa* based on the scientific literature.

## 2. Botanical Characterization and Distribution

*I. suffruticosa* is described as a shrub plant, measuring 1 m to 2 m height, having branches pubescent, stem angular, of grayish color, pinnate leaves composed of 7–15 oblong or oval leaflets, hairless on the face and back, with small flowers, numerous albo-pink or yellow, in axillary racemes, and its fruit is a small sickle pod with 6–10 seeds measuring 25 mm in length [13]. Having strong adaptability, they are considered wild plants that grow in all types of soils, tolerating drought, floods, and high salinities.

**Figure 1:**
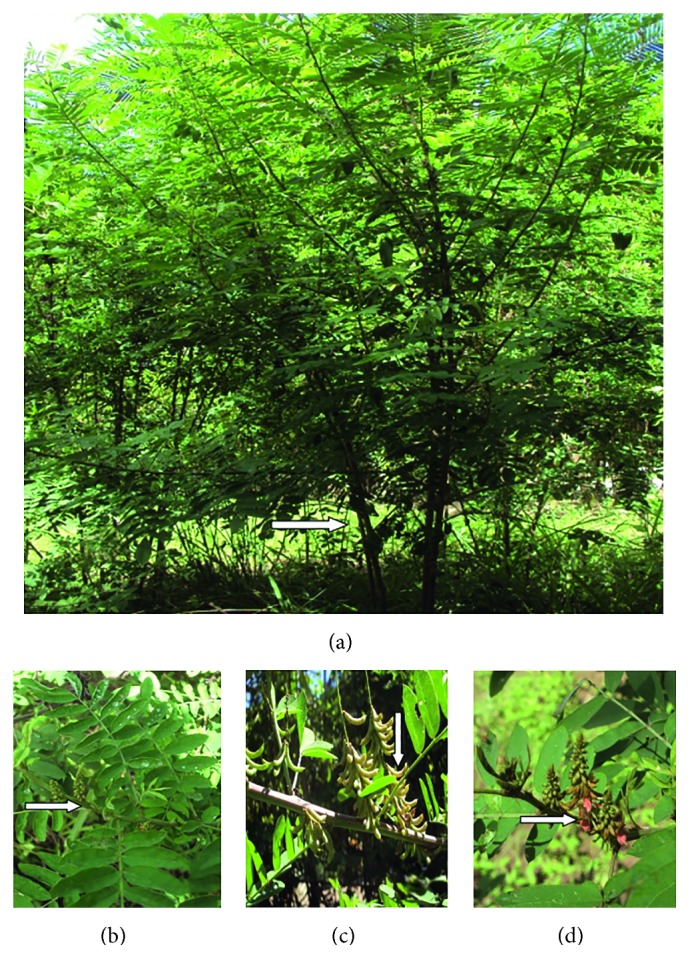
(a) Shrub *Indigofera suffruticosa* measuring approximately 1.15 m; (b) leaf and inflorescence; (c) branches with leaves and seeds; (d) branches with flowers, leaves, and inflorescence.

*I. suffruticosa* Mill. (Figure 1) is a species from the Antilla and Central America [14] and more prevalent throughout the tropical America. In Brazil, it is distributed in some states: São Paulo, Sergipe, Bahia, Rio de Janeiro, Minas Gerais, Maranhão [15, 16], Mato Grosso [17], Alagoas [4], Paraíba [18], Ceará, Rio Grande do Norte, Pernambuco, and Pará [19].

## 3. Traditional Use and Ethnopharmacology

*Indigofera suffruticosa* is popularly known as “indigo” or “anileira.” Such a nickname comes from the German language, meaning “blue pigment,” which is extracted through fermentation by hot infusion of its leaves and was used in the textile industries to dye yarns. Currently, this extract is processed by industrial chemical processes, and the use of this plant in the textile industries was abandoned [7]. *I. suffruticosa* may also be related to other popular names such as jiquilite, tzitzupu, indigo fields, anileira guinea, real anileira, caá-chica, caá-chira, timbó-mrim, timbozinho, and indigueira. The species is widely used in folk medicine to cure many health problems, with uses based on infusions and decoctions of different parts of this plant [20]. They are attributed to this plant's febrifuge, antispasmodic, diuretic, abortive, and analgesic properties against stomach and urinary problems, jaundice, ulcers and purgative, sedative, and insecticidal properties [21].

## 4. Chemical Constituents

Several studies have identified and isolated some chemical constituents of *I. suffruticosa*, including flavonoids, alkaloids, coumarins, triterpenoids, and carbohydrates. Early investigations of the chemical components of *I. suffruticosa* were made by Miller and Smith, 1973, using seed extract, with a highlight of the rich presence of amino acids and possible toxic effects. According to the Natural Products Alert [22] and Chemical Abstracts, the phytochemical profile of this species reveals the presence of alkaloids, polyphenols, terpenoids, steroids, reducing sugars, proteins, and indigoids.

Paiva et al. [23] quantified proteins and natural fibers of this species, showing its use as feed for ruminants. Isolation of 3-nitropropanoic acid glucose esters is featured by having toxic effects due to its conversion to the 3-nitropropanoic acid, a respiratory toxin that inhibits mitochondrial enzymes [24, 25]. In addition, *D*-(+)-pinitol, *β*-sitosterol, and louisfieserone have been isolated from this plant [25]. Apart from these isolated compounds, Kamal and Mangla [26] identified, characterized, and quantified six rotenoids from different parts of *I. suffruticosa*. Preliminary studies of leaves, seeds, and stems of *I. suffruticosa* demonstrate the presence of polyphenols (coumarin and chlorogenic acid) and flavonoids (quercetin, rutin, and gallic acid), alkaloids, triterpenoids, and carbohydrates [9, 27].

The main flavonoids identified and isolated from the methanol extract of *I. suffruticosa* leaves include quercetin 7-O-*β*-*d*-glucopyranoside, quercetin 3-O-[*β*-*d*-xylopyranosyl-(1⟶2)-*β*-*d*-galactopyranoside], quercetin 3-O-[*α*-*l*-rhamnopyranosyl-(1⟶6)-*β*-*d*-glucopyranoside], and quercetin 3-O-[*β*-*d*-glucopyranosyl-(1⟶2)-*β*-*d*-glucopyranoside. In addition to these compounds, indigo and indirubin were also isolated [27].

Pentadecanoic acid, 14-methyl-, *n*-hexanedecanoic acid, *z*-[13, 14-epoxy]tetradec-11-en-1-ol acetate, oleic acid, 9-octadecenoic acid[*z*]-, 2-hydroxy-1-[hydroxyl methyl], heptanoic acid, docosyl ester, octadecanoic acid, 7-hydroxy-, 6-octadecenoic acid[*z*]-, and 8-octadecenoic acid [28] were also found.

Chen et al. [29] using aqueous and ethanol extracts of *I. suffruticosa* identified the following different phenolic compounds: syringic acid, p-coumaric acid, vanillin, syringaldehyde, salicylic acid, quercetin, isoliquiritigenin, and formononetin.

The presence of such compounds in the leaf oil of *I. suffruticosa*, (*z*)-3-hexenyl benzoate, methyl hexadecanoate, phytol, linoleic acid, methyl linoleate, *n*-docosane, *n*-tricosane, was also found [30].

## 5. Pharmacological Activities

### 5.1. Embryotoxic and Cytotoxic Activity

Leite et al. [10] investigated the cytotoxic potential of aqueous extracts from leaves of *I. suffruticosa* in mouse embryos and found that, at high concentrations of the extract, the growth of the embryos was inhibited, preventing them reaching the final stage of embryogenesis, indicating that their use in high doses in humans can be harmful.

Vieira et al. [8] realized in his studies that aqueous extracts of leaves of *I. suffruticosa* by infusion and maceration in different concentrations (from 6.25 to 50 *µ*g/ml) tested in cell lines of HEp-2 by the MTT method did not produce any cytotoxic effect (>30 *µ*g/ml) when compared with the control and DMEM (Dulbecco's Modified Eagle Medium).

In another study, the indigo ethanol extract purifier of *I. suffruticosa* showed a potent cytotoxic agent, showing the value of 0.89 for the breast tumor cell line (LM2) and lung tumor cell line (LP07), clarifying that the extract has cellular responses such as inducing apoptosis [31].

Carli et al. [32] also observed the cytotoxic effect on cell viability assays with 3-(4,5-dimethylthiazol-2-yl)-2,5-diphenyl-tetrazolium bromide (MTT), having inhibitory concentration (IC_50_) of 200 *μ*g/ml.

Bioactive compounds of natural origin are essential in antineoplastic therapy, since they show promising effects on carcinogenesis and contribute to new medicinal interests. Therefore, such information has great relevance in understanding the use of this species and its promising future.

Vieira et al. [33] used aqueous *I. suffruticosa* extracts at varying doses (250–1000 mg/ml) in models for embryotoxicity in the development and oviposition of *Aedes aegypti*. In the study, a significant repellent effect was found on oviposition and an embryotoxicity was also observed, slowing the normal growth of the larvae of *Aedes aegypti*.

The use of plant extracts is an alternative method for insect control, which can minimize the harmful effects of some insecticides on nontarget insect species, humans, and the environment, leading to new opportunities for the development of commercial products of natural origin and of great importance.

### 5.2. Antitumor Activity

Vieira et al. [8] evaluated the effect of aqueous extracts of *I. suffruticosa* processed by maceration and infusion at a dose of 50 mg/kg on sarcoma 180 and found that both forms had significant effect on reducing the tumor (62.6 and 64.5%), respectively.

Cancer is still the leading cause of death in the world, and plants used in traditional medicines may be a potential source of powerful chemopreventive agents because they are enriching source of beneficial secondary components. There are many plant species that have relevant biological data in the scientific literature and even commercial use for this purpose. However, the use of *I. suffruticosa* is still poorly studied, and new studies should investigate the possibility of using as an antitumor drug.

### 5.3. In Vivo Activity against Ectoparasite (*Pediculosis capitis*)

García et al. [34] used tincture of *I. suffruticosa* to 5% in the population reduction of *Pediculosis capitis* and observed the population reduction of lice mainly in cases of persistence in patients of 55 years. After 2 days of application, the results confirmed the insecticidal activity of *I. suffruticosa* tincture; this treatment seems to be a valuable and effective alternative to existing treatments.

### 5.4. Antimutagenic Activity

Calvo et al. [35] evaluated the effect of the ethanol extract of the aerial parts of *I. suffruticosa* in trials on Salmonella, which showed mutagenic activity, suggesting that such an action is due to the presence of indigo pigment. Since plants are primary food sources and cure, the natural products need to be evaluated with regard to their toxicity and dosage because the indiscriminate use of homemade preparations of plants can be dangerous to health.

### 5.5. Antioxidant Activity

Ethanolic extracts of leaves of *I. suffruticosa* stand out with potent antioxidant activity in an experimental model in vitro with the free radical 2,2-diphenyl-1-picrilidrazil (DPPH), and this method is a rapid way to measure the antioxidant capacity of compounds, it is based on the reduction of DPPH in solution, and this action is attributed to the presence of high concentrations of gallic acid in the extract [30]. Plants contain several phytochemicals, such as phenolic compounds, which have promising antioxidant activity, mainly in the prevention of chronic diseases.

### 5.6. Hepatoprotective Activity

The aqueous extract of *I. suffruticosa* (50 mg/kg, ip) showed the protective effect on liver tissue of mice bearing sarcoma 180 [36]. Lima et al. [37], using the purified indigo compound from the leaves of *I. suffruticosa*, did not observe a reduction in sarcoma 180 tumor but found that liver cells remained preserved, emphasizing its hepatoprotective effect.

Lima et al. [37] investigated the antitumor action of indica, a compound extracted from the leaves of *I. suffruticosa*, on mice bearing sarcoma 180 cell lines and found that such a compound did not promote a reduction in tumor growth; however, it is found that treatment with indica does not allow to alter the architecture of the liver cells, suggesting a hepatoprotective effect.

Such an effect is considered of extreme importance since liver diseases have become a global public health problem, and much of them are a consequence of the prolonged use of chemicals and drugs.

### 5.7. Antimicrobial Activity

Santos et al. [38] evaluated the antimicrobial activity of ether, chloroform, and acetone extracts of *I. suffruticosa* against nine strains of *Staphylococcus aureus*, with minimal inhibitory concentration (MIC) ranging from 0.78 to 6.25 mg/ml. The methanol extract of the aerial parts of *I. suffruticosa* showed a significant effect against *Mycobacterium tuberculosis* with an MIC of 125 *μ*g/mL, suggesting the presence of an important bactericidal agent [32].

The endophytic fungi isolated from *I. suffruticosa* also showed activity against different bacteria such as *B. subtilis*, *S. aureus*, *E. coli*, *K. pneumoniae*, and *P. aeruginosa*, with an MIC ranging from 0.39 to 6.25 mg/ml, emphasizing that this species has a potential to inhibit bacterial growth [39].

The aqueous extract of the leaves of *I. suffruticosa* displays significant results against two strains of *Trichophyton rubrum* and *Microsporum canis*, with concentrations of 5 and 10 mg/ml with variation in MIC between 20 and 15 mm, with a similar effect to the standard drug ketoconazole (MIC −20 mm) [11].

The results obtained in these studies suggest that the extracts used had bioactive compounds responsible for the efficacy of the organic material studied. Not only the plant extracts but also those produced by microorganisms can be a source for the industrial manufacture of drugs and useful in the therapy of some microbial infections.

### 5.8. Anticonvulsant Activity

The *I. suffruticosa* fluid extract (0.06 g/kg for 10 days) in experimental models of shock promotes protective effect in both doses administered orally or intraperitoneally on seizures induced by this model, highlighting the antiepileptic potential of such an extract [40].

At the concentration of 0.06 g/kg, the fluid extract of *I. suffruticosa* was tested in models of chronic epilepsy, which reduced the concentration of inhibitory amino acids (glycine and tannin) and increased the excitatory amino acid (glutamic acid) [41].

In models of seizures, the methanol extract of the leaves of *I. suffruticosa* showed significant activities, and its action is due to the presence of secondary metabolites such as flavonoids and linalool having an action on the GABAergic system [42].

The anticonvulsant effect of the aqueous extract of *I. suffruticosa* was confirmed following behavior and electrophysiological analyses in rats. The systematic analysis of seizure behavior and its potentials was also recorded [36].

The findings of these studies stimulate the continuity of research in the search for new treatments for epilepsy. Although traditional therapy has good efficacy, it has high toxicity, and about 20–30% of patients who use this therapy are unable to control their seizures appropriately and have severe side effects. Therefore, new alternatives that reduce clinical manifestations and chronic conditions become essential.

### 5.9. Gastroprotective Activity

Luiz-Ferreira et al. [43] explored the gastroprotective effect of chloroformic and methanolic extracts of the aerial parts of *I. suffruticosa*, partitioned with ethyl acetate and administered at a dose of 100 mg/kg, which significantly inhibited the gastric mucosal lesions induced by ethanol and nonsteroidal drugs in rats.

In traditional medicine, several plants are used in the treatment of gastric disorders, and from the evidence presented here, it could be stated that extracts obtained from the aerial parts of *I. suffruticosa* are interesting sources for the development of a phytotherapeutic formulation to treat gastric ulcer.

### 5.10. Immunostimulatory Activities

Carli et al. [32] investigated the effect of the ethanol extracts of *I. suffruticosa* on immune activity *in vitro* and observed that the extract triggered a high nitric oxide production and stimulated the synthesis and release of TNF-*α*, thus triggering the activation of macrophages and promoting the production of other molecules that improve or restore the responsiveness of the innate immune system reaction against infections.

In another study, using a purified compound of *I. suffruticosa*, the indigo, also in experimental models *in vitro*, showed an increase in the production and release of nitric oxide and TNF-*α* [31].

The immune system is of fundamental importance to every living being, and its malfunction can cause various biological damages. Drugs that stimulate the production and action of the components of this system are of great biological relevance, primarily in immunodeficient individuals who are more susceptible to infections. In this way, the good results obtained for this species favor even more effective use in several diseases.

### 5.11. Anti-Inflammatory Activity

Aqueous extracts of *I. suffruticosa* (250 mg/kg) in inflammation experimental models of mice showed significant anti-inflammatory effect, with similar action to the commercial standard drug, acetyl salicylic acid [9].

In another study, aqueous and ethanolic extracts of leaves of *I. suffruticosa* were used in experimental models of inflammation induced by lipopolysaccharides (LPS) in macrophages, and it was possible to observe a significant anti-inflammatory effect [29].

The prolonged use of anti-inflammatories promotes several biological damages and the search for new drug therapies that reversibly abuse the harmful effect, and the low cost is still incessant. The studies already published show that *I. suffruticosa* is a strong candidate to be applied in this activity.

### 5.12. Gynecological Problems/Issues

Yazbek et al. [44] reported that, in Brazilian folk medicine, leaves and root of *I. suffruticosa* have been commonly used to prepare tea for inflammatory diseases of the gynecological tract, mainly organs such as ovaries and/or uterus.

In many places, the main therapeutic resource for the treatment of discomforts and diseases of gynecological origin is still commonly represented by medicinal plants. However, few studies, such as [44], show the promising effect that may resonate on new beneficial therapeutic approaches and on strengthening the practice of women's periodic care.

## 6. Toxicity

Studies using aqueous extracts obtained by infusing leaves of *I. suffruticosa* in acute toxicity in mice demonstrated the presence of deaths in the tested groups. Some signs of toxicity were noted after a few hours of intraperitoneal administration from a lower concentration to a higher concentration tested dose (2400 mg·kg^−1^): agitation, piloerection, exhaustion, sleepiness, irritability, and spasms. Also, it was found that the LD50 of the acute toxicity of the aqueous extract of leaves of *I. suffruticosa* made by infusion administered in different doses in mice showed no mortality during 72 h of observation [33].

The methanol extract of leaves of *I. suffruticosa* showed a low toxicity with an LD50 of 1600 mg/kg (ip) in mice. The results exhibit a lower significant change in individual behavioral and parameters that slight decrease in spontaneous locomotor activity and an increase in breathing frequency [42].

## 7. Conclusions

Plants since ancient times have been used as medicine and have been daily providing inspiration for new research, aiming to highlight the diverse potential and expand the library of biologically active molecules.

In recent years, *I. suffruticosa* has attracted the attention of many researchers because of its high therapeutic value in the population. Different extracts of this species have presented significant results in several pharmacological activities, such as antitumor, antioxidant, anti-inflammatory, antimicrobial, antiepileptic, antifungal, anticonvulsant, gastroprotective, and hepatoprotective.

These studies were tested *in vivo* in laboratory animals, and the results presented are not enough for humans to use. Based on the low toxicity presented by the statement and little research of their phytochemicals, new clinical trials should be conducted to fill the gaps in research to establish baseline data for medicinal use, eliminating potential harmful risks and promoting beneficial effects.

## References

[1] Oliveira D. M. T., Paiva E. A. S. (2005). *Pterodon emarginatus* (Fabaceae: Faboideae) seed. *Brazilian Journal of Biology*.

[2] Dutra V. F., Messias M. C. T. B., Garcia F. C. P. (2005). Papilionoideae (Leguminosae) dos campos ferruginosos do Parque Estadual do Itacolomi, MG, Brasil: florística e fenologia. *Revista Brasileira de Botânica*.

[3] Ferreira P. S. M., Trovão D. M. D. B. M., Melo J. I. M. D. (2015). Leguminosae at APA do Cariri, Paraíba State, Brazil. *Hoehnea*.

[4] Ribeiro I. M., Silva M. A., Rangel J. H. A. R. (1984). *Levantamento Botânico de Leguminosas Forrageiras Nativas da Bacia Leiteira do Estado de Alagoas*.

[5] Sherman P. J. (1982). *Tropical Forage Legumes*.

[6] Froman B. (1975). *Na Illustrateg Guide to the Pasture Legumes of Ethiopia*.

[7] Pesavento F. (2005). O azul fluminense: um estudo sobre o comércio do anil no Rio de Janeiro Colonial. *Revista Econômica*.

[8] Vieira J. R. C., Souza I. A., Nascimento S. C., Leite S. P. (2007). *Indigofera suffruticosa*: an alternative anticancer therapy. *Evidence-Based Complementary and Alternative Medicine*.

[9] Leite S. P., Silva L. L. S., Catanho M. T. J. A., Lima E. O., Lima V. L. M. (2003). Atividade anti-inflamatória do extrato de *Indigofera suffruticosa*. *Revista Brasileira de Ciências da Saúde*.

[10] Leite S. P., Medeirosa P. L., Silva E. C., Maia M. B. S., Lima V. L. M., Sauld D. E. (2004). Embryotoxicity *in vitro* with extract of *Indigofera suffruticosa* leaves. *Reproductive Toxicology*.

[11] Leite S. P., Vieira J. R. C., Medeiros P. L. (2006). Antimicrobial activity of *Indigofera suffruticosa*. *Evidence-Based Complementary and Alternative Medicine*.

[12] Roig T., Mesa J. T. (1974). *Plantas Medicinales Arómaticas y Venenosas de Cuba*.

[13] Braga R. (1976). *Plantas do Nordeste Especialmente do Ceara*.

[14] Almeida E. R. (1993). *Plantas Medicinais Brasileiras: Conhecimentos Populares e Científicos*.

[15] Moreira J. L. A., Azevedo-Tozzi A. M. G. (1997). *Indigofera* L. (Leguminosae, Papilionoideae) no estado de São Paulo, Brasil. *Revista Brasileira de Botânica*.

[16] Mioto S. T. S., Iganci J. R. V. (2015). *Indigofera in: Lista de Espécies da Flora do Brasil*.

[17] Fernandes A. (1987). *Noções de Toxicologia e Plantas Tóxicas*.

[18] Riet-Correa F. (2000). *Comunicação Pessoal*.

[19] Neto J. D. B., Oliveira C. M. C., Barbosa I. B. P., Peixoto P. V., Ávila S. C., Tokarnia C. H. (2001). Anemia hemolítica causada por *Indigofera suffruticosa* (Leg. Papilionoideae) em bovinos. *Pesquisa Veterinária Brasileira*.

[20] Matos F. J. A. (1999). *Plantas da Medicina Popular do Nordeste: Propriedades Atribuídas e Confirmadas*.

[21] Hastings R. B. (1990). Medicinal legumes of Mexico: Fabaceae, papilionoideae, part one. *Economic Botany*.

[22] NAPRALERT (2015). *Natural Products Alert*.

[23] Paiva M. A. S., Barbosa A. C. D., Alves H. L. J. *Indigofera suffruticosa* Mill. (Leguminosae) com potencial forrageiro em uma região de Caatinga no Semi-árido de Pernambuco. (Alagoinha).

[24] Garcez F. R., Scramin S., Nascimento M. C., Mors W. B. (1988). Prenylated flavonoids as evolutionary indicators in the genus Dahlstedtia. *Phytochemistry*.

[25] Garcez W. S., Garcez F. R., Honda N. K., Silva A. J. R. (1989). A nitropropanoyl-glucopyranoside from *Indigofera suffruticosa*. *Phytochemistry*.

[26] Kamal R., Mangla M. (1993). In vivo, *in vitro*, Investigation on rotenoids from *Indigofera suffruticosa* and their bioefficacy against the larvas of Anopheles stephensi and adults of *Callosobruchus* chinesis. *Journal of Biosciences*.

[27] Calvo T. R. Uso sustentável de biodiversidade brasileira-prospecção químico-farmacológica em plantas superiores: *Alchornea glandulosa*, Alchorneatriplinervia (Euphorbiaceae), *Indigofera truxillensis* e *Indigofera suffruticosa* (Fabaceae).

[28] Vijisaral E. D., Arumugam S. (2014). GC-MS analysis of bioactive constituents of *Indigofera suffruticosa* leaves. *Journal of Chemical and Pharmaceutical Research*.

[29] Chen T. Y., Sun H. L., Yao H. T. (2013). Suppressive effects of *Indigofera suffruticosa* Mill. extracts on lipopolysaccharide-induced inflammatory responses in murine RAW 264.7 macrophages. *Food and Chemical Toxicology*.

[30] Arriaga A. M. C., Lemos T. L. G., Santiago G. M. P. (2013). Chemical composition and antioxidant activity of *Indigofera suffruticosa*. *Chemistry of Natural Compounds*.

[31] Lopes F. C. M., Calvo T. R., Colombo L. L., Vilegas W., Carlos I. Z. (2011). Immunostimulatory and cytotoxic activities of *Indigofera suffruticosa* (Fabaceae). *Natural Product Research*.

[32] Carli C. B. A., Quilles M. B., Maia D. C. G. (2010). Antimycobacterial activity of *Indigofera suffruticosa* with activation potential of the innate immune system. *Pharmaceutical Biology*.

[33] Vieira J. R. C., Leite R. M. P., Lima I. R., Navarro D. A. F., Bianco E. M., Leite S. P. (2012). Oviposition and embryotoxicity of *Indigofera suffruticosa* on early development of *Aedes aegypti* (Diptera: Culicidae). *Evidence-Based Complementary and Alternative Medicine*.

[34] García C. T., Rodríguez G. M. E., Pinera W. M. C., Martínez M. M. A., Santana S. Y., Hernández C. N. (2011). Effective treatment of a patient infested with *pediculus capitis* by using 5% *Indigofera suffruticosa* Mill. tincture. *Revista Cubana de Medicina Tropical*.

[35] Calvo T. R., Cardoso R. P. C., Moura A. C. S. (2011). Mutagenic activity of *Indigofera truxillensis* and *I. suffruticosa aerial* parts. *Evidence-Based Complementary and Alternative Medicine*.

[36] Silva I. B., Lima I. R., Santana M. A. N., Leite R. M. P., Leite S. P. (2014). *Indigofera suffruticosa* Mill. (Fabaceae): hepatic responses in mice bearing sarcoma 180. *International Journal of Morphology*.

[37] Lima I. R., Vieira J. R. C., Silva I. B., Leite R. M. P., Maia M. B., Leite S. P. (2014). Indican from Añil (*Indigofera suffruticosa* Miller) as an herbal protective agent to the liver. *Analytical and Quantitative Cytology and Histology*.

[38] Santos A. T. B., Araújo T. F. S., Silva L. C. N. (2015). Organic extracts from *Indigofera suffruticosa* leaves have antimicrobial and synergic actions with Erythromycin against *Staphylococcus aureus*. *Frontiers in Microbiology*.

[39] Santos I. P., Bezerra J. D. P., Sousa-Mota C. M., Cavalcanti M. S., Lima V. L. M. (2015). Endophytic mycobiota from leaves of *Indigofera suffruticosa* Miller (Fabaceae): the relationship between seasonal change in Atlantic Coastal Forest and tropical dry forest (Caatinga), Brazil. *African Journal of Microbiology Research*.

[40] Alejo J. L. P., Miranda R., Rodríguez G. (1996). Actividad anticonvulsivante (antiepileptica) delextracto fluido de *Indigofera suffruticosa* (AñilCimarron). *Revista Cubana de Plantas Medicinales*.

[41] Wong M. B., Rodríguez N. S., Alejo J. L. P., Pérez M. F. (1999). Actividad de la *Indigofera suffruticosa*millen la epilepsia crónica experimental y surelacióncon aminoácidos neurotransmisores. *Revista Cubana de Plantas Medicinales*.

[42] Almeida E. R., Chaves M. T., Luna R. L. A. (2013). Anticonvulsant effect of *Indigofera suffruticosa* Mill.: indication of involvement of the GABAergic system. *African Journal of Pharmacy and Pharmacology*.

[43] Luiz-Ferreira A., Cola M., Barbastefano V. (2011). *Indigofera suffruticosa* Mill. as new source of healing agent: involvement of prostaglandin and mucus and heat shock proteins. *Journal of Ethnopharmacology*.

[44] Yazbek P. B., Tezoto J., Cassas F., Rodrigues E. (2016). Plants used during maternity, menstrual cycle and other women’s health conditions among Brazilian cultures. *Journal of Ethnopharmacology*.

